# Imaging and Biomarkers: The Assesment of Pulmonary Embolism Risk and Early Mortality

**DOI:** 10.3390/medicina60091489

**Published:** 2024-09-12

**Authors:** Alexandru Gratian Naum, Irina Jari, Liliana Moisii, Andra Mara Ursu, Paloma Moisii

**Affiliations:** 1 2nd Morphofunctional Sciences Department, Biophysics and Medical Physics, “Grigore T. Popa” University of Medicine and Pharmacy, 16 Universitatii Street, 700115 Iasi, Romania; alexandru.naum@umfiasi.ro; 2 “Neolife” Medical Center, 52 Carol I Avenue, 700503 Iasi, Romania; 3 2nd Surgical Department, “Grigore T. Popa” University of Medicine and Pharmacy, 16 Universitatii Street, 700115 Iasi, Romania; liliana.moisii@umfiasi.ro; 4 St. Spiridon Emergency Hospital, Radiology and Medical Imaging Clinique, 1st Independentei Avenue, 700111 Iasi, Romania; andra-mara_ursu@email.umfiasi.ro; 5 1st Medical Department “Grigore T. Popa” University of Medicine and Pharmacy, 16 Universitatii Street, 700115 Iasi, Romania; 6 Promedicanon “Cardiology Office”, 15 Prisacii Valley, 707410 Valea Lupului, Romania

**Keywords:** pulmonary embolism, pulmonary artery obstruction index, D-dimer, cardiac troponin T

## Abstract

*Background and Objectives*: Pulmonary embolism (PE) incidence has been increasing in the last 10 years. Computed thoracic pulmonary angiography (CTPA) had a major role in PE diagnosis and prognosis. The main purpose of this study was as follows: the prognostic value of a CTPA parameter, pulmonary artery obstruction index (PAOI), in PE risk assessment and the predictive accuracy of biomarkers, D-dimer and cardiac Troponin T (c-TnT), in 7-day mortality. A second objective of the research was to investigate the relationship between imaging by PAOI and these biomarkers in different etiologies of PE. *Materials and Methods*: This study comprised 109 patients with PE, hospitalized and treated between February 2021 and August 2022. They had different etiologies of PE: deep vein thrombosis (DVT); persistent atrial fibrillation (AF); chronic obstructive pulmonary disease (COPD) exacerbation; COVID-19; and cancers. The investigations were as follows: clinical examination; D-dimer testing, as a mandatory method for PE suspicion (values ≥500 µg/L were highly suggestive for PE); c-TnT, as a marker of myocardial injury (values ≥14 ng/L were abnormal); CTPA, with right ventricle dysfunction (RVD) signs and PAOI. Treatments were according to PE risk: systemic thrombolysis in high-risk PE; low weight molecular heparins (LWMH) in high-risk PE, after systemic thrombolysis or from the beginning, when systemic thrombolysis was contraindicated; and direct oral anticoagulants (DOAC) in low- and intermediate-risk PE. *Results*: PAOI had a high predictive accuracy for high-risk PE (area under curve, AUC = 0.993). D-dimer and cTnT had a statistically significant relationship with 7-day mortality for the entire sample, *p* < 0.001, and for AF, *p* = 0.0036; COVID-19, *p* = 0.003; and cancer patients, *p* = 0.005. PAOI had statistical significance for 7-day mortality only in COVID-19, *p* = 0.045, and cancer patients, *p* = 0.038. The relationship PAOI–D-dimer and PAOI–c-TnT had very strong statistical correlation for the entire sample and for DVT, AF, COPD, and COVID-19 subgroups (Rho = 0.815–0.982). *Conclusions*: PAOI was an important tool for PE risk assessment. D-dimer and c-TnT were valuable predictors for 7-day mortality in PE. PAOI (imaging parameter for PE extent) and D-dimer (biomarker for PE severity) as well as PAOI and c-TnT (biomarker for myocardial injury) were strongly correlated for the entire PE sample and for DVT, AF, COPD, and COVID-19 patients.

## 1. Introduction

PE is usually suspected when the patient has dyspnea, chest pain, syncope, or hemoptysis [1]. Sudden onset of the dyspnea is highly suggestive for PE but is not a typical symptom. Bronchopneumonia and acute heart failure can have this onset. Chest pain must be differentiated by other life-threatening conditions, like aortic dissection and acute coronary syndromes. Syncope can also occur in acute cerebrovascular events, and the PE diagnosis becomes more difficult in geriatric patients with long-term immobility, and syncope. Hemoptysis is a common sign in PE, but pulmonary tuberculosis/pulmonary cancer can present with this sign.

PE diagnosis was frequently missed or delayed in the past, when the diagnosis algorithm included the clinical examination, electrocardiogram, and echocardiography. The probability of underdiagnosed PE was high utilizing only these methods. CTPA became the gold standard for PE diagnosis and increased accessibility for CTPA enhanced the true positive diagnosis of the disease. The last decade brought us new insights about PE. Earlier diagnosis and a modern therapeutic approach decreased mortality rates from 20% to 1–3% [2]. 

There are two major types of PE: isolated PE and DVT-associated PE.

For isolated PE, thrombus is formed in situ due to a local process or an embolus, which is formed elsewhere, arrives in the pulmonary arteries. Isolated PE is recorded as 50% of total cases of PE [3]. Rarely, ethiopathogeny can be factor V Leiden or prothrombin mutation (inherited thrombophilia) or antiphosholipidic syndrome (acquired thrombophilia). Very rarely, antithrombin, protein C, or S deficiencies can be implicated [4]. Even procoagulant status drives PE in these types of isolated PE; provoking factors are usually necessary: recent surgery or immobilization [5]. 

Respiratory diseases, like chronic bronchial asthma and chronic obstructive pulmonary disease (COPD), can be a frequent etiology for isolated PE. Around 30% of COPD patients associate isolated PE with in situ pulmonary thrombosis. Especially in acute exacerbations of COPD, PE screening is mandatory [6]. 

Cardiovascular diseases like myocardial infarction (MI), AF, coronary artery disease (CAD), and left-sided cardiomyopathy (CMP) can be another frequent etiology for isolated PE [7]. Both respiratory and cardiovascular diseases, previously mentioned, exhibit high levels of proinflammatory proteins. Oxidative stress determines upregulation of inflammation and in situ thrombosis [8]. In cardiovascular diseases, in situ thrombosis can be inside the heart chambers, with successive embolism in pulmonary arteries. Also, in situ thrombosis for PE related to cardiovascular diseases, can be inside pulmonary arteries from the beginning due to upregulation of inflammation, as previously described. 

In COVID-19 patients, the virus itself can provoke pulmonary microvascular dysfunction, followed by local thrombosis and PE [9]. This complication in COVID-19 patients increases exponentially the mortality [10]; de Godoy et al. noticed that 58% of COVID-19 patients with PE died [11].

For DVT-associated PE, an embolic event is produced. The thrombus is formed inside a deep vein (usually inferior limbs) and arrives in the pulmonary artery tree through the bloodstream. The most common risk factors for DVT are the following: obesity, which increases fibrinogen levels and provokes mechanical vein compression [12]; immobility, which reduces blood flow [13]; surgery, which provokes mechanical injury of the vein [14]; cancer, which creates hypercoagulable state [15]; and previous DVT.

D-dimer levels are elevated in PE because of simultaneous activation of coagulation and fibrinolysis [16]. Common medical conditions such as infections, cancer [17], and pregnancy [18] can increase D-dimer levels. PE diagnosis in these conditions is a challenge for the clinicians. Cut-off values for this biomarker are necessary for a correct diagnosis, especially in these situations. Specific medical conditions like intracerebral/subarachnoid hemorrhages and aortic dissection have elevated D-dimer levels. In these diseases, diagnosis trap with PE can be avoided with a complete evaluation of the patient. D-dimers are important biomarkers for PE diagnosis, but clinical judgement and complementary methods can clarify the diagnosis. Normal levels of D-dimer make PE diagnosis unlikely (high negative predictive value). Conversely, the positive predicted values of D-dimer testing are low [19].

Elevated markers of myocardial injury (cardiac troponins) are noticed in 30–60% of PE patients. The highest percentages (60%) are seen in high-sensitivity assays for troponins. When conventional assays are utilized for troponin concentration in PE, the percentages diminish around 30% [20]. A worse prognosis may be revealed in patients with elevated cardiac troponins in PE [21].

CTPA is the gold standard for PE diagnosis. The major criterion for PE is endoluminal filling defect on CTPA.The following parameters are collected by CTPA: 1. ratio between the transversal diameter of the right ventricle and left ventricle (RV/LV); 2. ratio between the transversal dimension for the pulmonary artery trunk and aorta (PAT/Ao); 3. septal deviation; 4. contrast reflux in the inferior vena cava; and 5. PAOI or Qanadli score. The first four CTPA parameters are utilized for right ventricle dysfunction (RVD) assessment [22]. PAOI is a percentage which reflects what part of the pulmonary arteries’ tree is obstructed by the thrombus. The percentage of vascular obstruction was calculated through the following: dividing the patient score (nxd) by the maximal total score (40) and by multiplying the result with 100. Thereby, PAOI is calculated by this formula: embolism number (n) × embolism degree (d)/40 × 100%. Embolism number (n) is the value of the proximal thrombus site, and this is equal to the number of segmental pulmonary arteries arising distally to the thrombus site. Values from n range from a minimum of 1 (one segment obstructed) to a maximum of 20 obstructed arteries. Embolism degree (d) is 1 for partial obstruction and 2 for total obstruction [23].

Risk assessment in PE can be stratified by specific investigations: elevated cardiac troponin levels and RVD on CTPA or on transthoracic echocardiography [24].

The main objective of our study was as follows: the prognostic value of PAOI for risk assessment in PE and the predictive accuracy of D-dimer, c-TnT, and PAOI for 7-day mortality in PE. The relationship between imaging (PAOI) and biomarkers (D-dimer and cTnT) in different etiologies of PE was a secondary objective of the study.

## 2. Materials and Methods

### 2.1. Study Design

This study was conducted in accordance with the rules and principles of evidence-based medicine, in compliance with the requirements of the Declaration of Helsinki of the World Medical Association 2013, and was approved by the Committee of Ethics of ‘’Saint Spiridon” Emergency Hospital, Iasi, protocol no. 42, dated 27 May 2020.

The recruitment of patients, the investigations, and their treatment was completed in ‘’St. Spiridon” Emergency Hospital, from February 2021 to August 2022. The research type was a retrospective cross-sectional study. The study comprised 109 patients diagnosed with acute PE. The entire sample (n = 109) was divided in 5 groups according to PE etiology. The first group included 35 patients with DVT-associated PE. The other four groups included patients with isolated PE. The second group comprised 32 patients with permanent AF; the third group had COVID-19 pneumonia as PE etiology and included 28 patients. The fourth group had 14 patients with cancer and the fifth group comprised 7 patients with acute exacerbation of COPD.

Multiple etiology for PE was noticed in 7 of 109 patients. Six patients had double etiology for PE: three patients with cancer also had DVT; in two patients with COVID-19, one also had cancer and the other one AF; and one patient with meningitis also had AF. One patient had triple etiology for PE: AF, COVID-19, and cancer.

### 2.2. Inclusion Criteria

To be included in the study, the diagnosis and the treatment protocol were explained with each patient. An informed consent approved by the ethics comitee was signed. For patients with hemodynamic instability, this consent was signed after we managed the emergency treatment. Age above 18 years and 7-day follow up during hospitalization were mandatory inclusion criteria. 

### 2.3. Methods

- Clinical examination was focused on dyspnea, hemoptysis, heart failure signs (dilation of jugular veins; cyanosis; tachycardia; hepatomegaly; hepatic jugular reflux; and cardiac edema), blood pressure, body mass index calculation (BMI = weight (in kilograms)/height^2^ (squared meters)). Obesity was defined if BMI was over 30 kg/m^2^.
- CTPA was performed with a Philips Incisive 64 detector CT scan with the following protocol:
  - Non-contrast enhanced scan;
  - The arterial phase scan delay was 7 s after the density in the pulmonary artery reached 180 HU (Hounsfield units);
  - The venous phase at 20 s after the arterial phase.

The following parameters were collected by CTPA and were useful for RVD assessment: 1. RV/LV (values above 1 were sugestive for RVD); 2. PAT/Ao (a value above 1 was a CT sign for RVD); 3. septal deviation (its presence was suggestive for RVD); and 4. contrast reflux in inferior vena cava (its presence was a CT sign for RVD). PAOI was a major CTPA parameter, which split the sample into minor PE if PAOI < 32.5% and massive PE if PAOI ≥32.5%. The cut-off value for D-dimer resulted from statistical analysis.

c. D-dimer testing utilized Fiatest AFT-300, CTK Biotech, Inc., Poway, CA, USA. The quantitative enzyme-linked immunosorbent assay (ELISA) was made in the emergency department. D-dimer values > 500 µg/L were considered abnormal.
d. Cardiac troponin testing utilized Fiatest AFT-300, ELISA technique, and high-sensitivity assay. The C-TnT cut-off value was above 14 ng/L.
e. Risk stratification and protocol treatment were utilized.

Risk stratification was calculated according to European Society of Cardiology guidelines. Hemodynamics and right ventricle function were the most important parameters for the risk assessment. High-risk PE has RVD, elevated c-TnT, and hemodynamic instability. Intermediate-risk PE has RVD and elevated c-TnT. Low-risk PE has none of the previously described findings. Treatment recommendations were made in concordance with risk stratification [25,26].

Protocol treatment for PE was represented by the following medication in our research:

- Systemic thrombolysis, with recombinant tissue plasminogen activator (rTPA) Alteplase 100 mg in 2 h, via intravenous administration, in high-risk PE;
- LWMH Enoxaparine 1 mg/kg twice daily, via subcutaneous injection, for the first week (in high-risk PE after systemic thrombolysis or from the beginning in high-risk PE if systemic thrombolysis was contraindicated); after the first week, LWMH were followed by DOAC;
- DOAC, Apixaban 10 mg twice daily for the first 7 days in low- and intermediate-risk PE, via oral administration. After the first week, the patients received Apixaban 5 mg twice daily or 2.5 mg twice daily. Apixaban 2.5 mg twice daily was given in the following circumstances: age ≥ 80 years, weight ≤ 60kg, and creatinine values > 1.5 mg/dL. Apixaban was contraindicated in gastro-intestinal cancer, so these patients received LWMH.

The duration of anticoagulation was 6 months for reversible conditions for PE (DVT, COVID-19, and COPD exacerbations). Long-term administration was necessary in chronic and irreversible conditions for PE (persistent AF and cancer with metastasis).

### 2.4. Statistical Analysis

SPSS 29.0 was the software for statistical analysis. For the D-dimer–PAOI relationship, we used Mann–Whitney test (*p* value < 0.05 indicated statistical significance, while *p* < 0.01 indicated high statistical significance) and Spearman’s correlation coefficient (Rho; 0.8 ≤ Rho ≤ 1 signified a very strong statistical correlation). For the c-TnT–PAOI relationship, we used Pearson chi-squared test (*p* value < 0.05 indicated statistical significance, while *p* < 0.01 indicated high statistical significance) and Spearman’s correlation coefficient, Rho (0.8 ≤ Rho ≤ 1 defined a very strong statistical correlation). AUC and receiver operating characteristic curve (ROC) analyzed prediction accuracy of PAOI for high-risk PE assessment. Pearson chi-squared test was also utilized for the relationship between D-dimer, c-TnT, PAOI, and 7-day mortality in PE (*p* value < 0.05 indicated statistical significance, while *p* < 0.01 suggested high statistical significance).

## 3. Results

### 3.1. The Clinical Characteristics of the Entire Sample

Females were predominant in our study (55%). The mean age was 66.79 ± 14.017 years (range, 28–93 years). Obesity was noticed in 62 of 109 patients (58.7%). Even if obesity had a high prevalence in our research, this comorbidity was not associated with mortality. Dyspnea was the most common symptom in the study: 73 of 109 patients (66.9%) revealed this complaint. Right-sided heart failure signs (during clinical examination) were noticed in 40.3% of our patients. These clinical signs were the following: dilation of jugulair veins; cyanosis; tachycardia; hepatomegaly; hepatic jugular reflux; and cardiac edema. Hemodynamic instability at admission was frequent: 38 of 109 patients (34.9%) had systolic blood pressure <90 mmHg. Cardiogrenic schock had 12 of these 38 patients with arterial hypotension. The most frequent etiology for PE was DVT (32.1%), followed by persistent AF (29.4%). A quarter of the sample had COVID-19 etiology for PE (25.7%). Pulmonary cancer, malignant lymphomas, pancreatic/rectal/breast, and uterine cancers were the neoplastic diseases with PE in our study (12.8%). COPD exacerbations had the lowest percentage in PE etiology (6.4%). All these results are summarized in Table 1. 

RVD was revealed by CTPA on 46 of 109 patients (42.2%). The percentage of imaging RVD patients was close to the percentage of right-sided heart failure clinical signs (40.3%). Minor PE was noticed in 73 of 109 patients (67%) and massive PE in 36 of 109 patients (33%). The cut-off value for PAOI was 32.5%; PAOI < 32.5% defined minor PE and PAOI ≥ 32.5% in CTPA defined massive PE. 

### 3.2. The Correlations between Biomarker Values at Admission (D-Dimer and c-TnT) and PAOI

In minor PE patients, the highest value for D-dimer was noticed in cancer etiology (median = 923 µg/L), and the lowest value for D-dimer was revealed in AF etiology (median = 711 µg/L). In massive PE patients, the highest value for D-dimer has been recorded in COVID-19 etiology (median = 1493 µg/L) and the lowest value in DVT etiology (median = 1257 µg/L).

The correlation between PAOI and D-dimer had high statistical significance for the entire sample and for DVT, AF, and COVID-19 etiologies, with *p*-value < 0.001. For COPD and cancer etiologies, this correlation did not have statistical significance, with *p*-value = 0.095 for COPD and 0.352 for cancer patients in the relationship PAOI–D-dimer. A very strong statistical correlation (Rho) was observed for PAOI and D-dimer for the entire sample (Rho = 0.855) and for each subgroup (DVT: Rho = 0.908; AF: Rho = 0.942; COPD: Rho = 0.982; COVID-19: Rho = 0.913), with one exception: cancer etiology (Rho = 0.091). In cancer etiology, the severity of PE was not corelated with D-dimer levels. In cancer patients, elevated D-dimer levels were not associated with higher PAOI, like in DVT, AF, COVID-19, and COPD etiologies. These results suggested that cancer patients with PE had elevated D-dimer levels also in the context of cancer and not due only to PE (as for other etiologies). The results are illustrated in Table 2.

C-TnT, the biomarker for myocardial injury, was increased in 50.4% of the patients (33% with massive PE and 17.4% with minor PE). The correlation between PAOI and c-TnT had high statistical significance for all the sample and for DVT, AF, and COVID-19 etiologies, as with the correlation between PAOI and D-dimer: *p* < 0.001. In COPD and cancer etiologies, the correlation PAOI–cTnT had no statistical significance: *p* = 0.110. Rho revealed a very strong correlation between PAOI and cTnT for the entire sample (Rho = 0.815) and for each etiology (Rho = 0.882 in DVT, Rho = 0.937 in AF, Rho= 0.982 in COPD, and Rho = 0.828 in COVID-19 patients). For the cancer group, the PAOI and c-TnT correlation had no statistical significance: *p* = 0.110 and Rho = 0.118. In cancer patients, these results suggested that myocardial injury is not more severe when PAOI is higher, like in other etiologies. Elevated c-TnT values were detected on advanced stages of cancer, and these elevated values were not related with PE.The results are summarized in Table 3.

### 3.3. PE Risk Predicted by PAOI and Mortality Assesment by D-Dimer and c-TnT Values

High-risk PE was noticed for 33% of the patients and they received systemic thrombolysis. The DVT patients had the highest percentage for high risk among all the patients (11.9%).

The four patients with intermediate-risk PE had minor PE at CTPA, and their PE etiology was COPD exacerbation. The results about PE risk assesment are summarized in Table 4.

**Figure 1:**
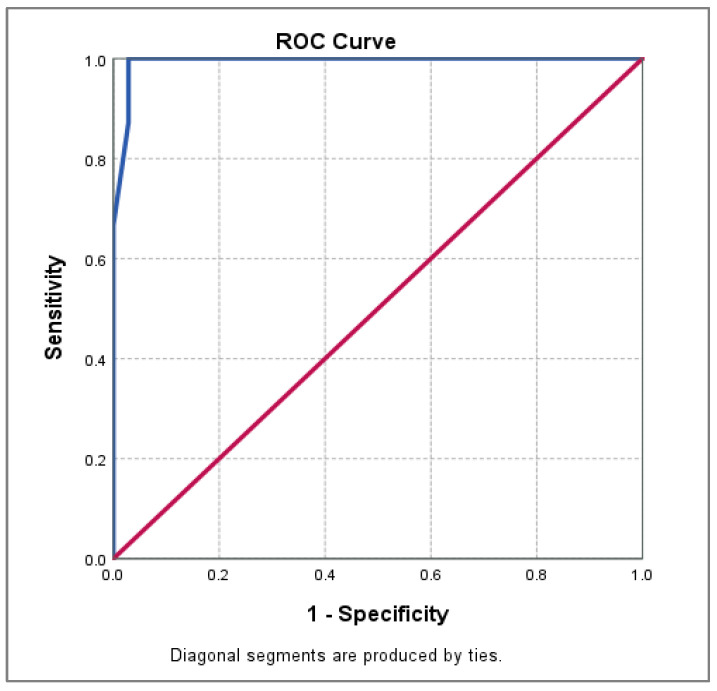
ROC curve for PAOI in high-risk PE prediction.

We investigated PAOI predictive accuracy in PE risk. For high-risk PE, the PAOI cut-off value was 32.5% and AUC = 0.993 (very high accuracy). The sensitivity was 100% and specificity was 97.1%. PAOI was an accurate predictor for high-risk PE. Table 5 and Figure 1 illustrate these results.

The mortality rate was 8.25% (9 of 109 patients). In two deceased patients with AF (1.82%), one of them also had COVID-19 and the other one meningitis. One deceased patient with DVT (0.91%) also had cancer, a malignant lymphoma. Among COVID-19 patients, 5 of 28 were deceased (4.58%). Four deceased patients from the COVID-19 group had massive PE at CTPA. There were two deaths among cancer patients (1.82%); they had pulmonary, cerebral, and hepatic metastasis. Among the nine deceased patients, they had massive PE with COVID-19 (3.66%)/double etiology of PE (2.73%)/metastatic cancer (1.82%).

D-dimer, and cTnT had a statistically significant relationship with 7-day mortality for the entire sample, *p* < 0.001, and for AF, *p*= 0.0036; COVID-19, *p* = 0.003; and cancer patients, *p* = 0.005. PAOI and 7-day mortality had statistical significance only in COVID-19, *p* = 0.045, and cancer patients, *p* = 0.038. We did not notice statistical significance for the relationship of PAOI and 7-day mortality in AF, *p* = 0.532, and for the entire sample, *p* = 0.741. Elevated biomarker (D-dimer and c-TnT) values were correlated with higher 7-day mortality in this study for the entire sample and for specific etiologies: AF, COVID-19, and cancer. These results are illustrated in Table 6, Table 7, Table 8 and Table 9.

## 4. Discussion

This study selected 109 patients, diagnosed with acute PE, with different etiologies. This is a peculiar aspect of the research because, usually, studies with PE present separately a specific cause (postoperative, long-term immobility, pregnancy, DVT, cancer, and infections). Our patients had DVT-associated PE and, from isolated PE, they had AF, COPD, cancer, and COVID-19 infection. We compared the prognostic value of biomarkers (D-dimer and c-TnT) and imaging (PAOI) in DVT-associated PE and in the other four causes of isolated PE.

The most important aspects of clinical examination were the following: obesity, dyspnea, right-sided heart failure signs, and hemodynamic instability.

Obesity had a high prevalence in our sample (58%), but it had no relevance for 7-day mortality. All the deceased patients had normal BMI in our research. Alkhalfan et al. concluded that obese patients had a lower risk of PE mortality, despite the higher prevalence of PE in obese than in nonobese patients [27]. This is called “the obesity paradox in PE”. Moreover, hemodynamic instability was a common condition in our obese patients, but this life-threatening situation was managed by inotropic agents and systemic thrombolysis. Obese patients with hemodynamic instability at admission did not have a higher risk for mortality in our research. Similar observations were noticed by Tamini et al.: an increased hemodynamic instability of obese patients with PE, without increased mortality rate [28].

Dyspnea was a common symptom in our patients, with a high prevalence (66.9%). This complaint was increased in COPD exacerbations and in COVID-19 patients, but we investigated the PE risk. We noticed the following relationship between dyspnea at admission and PE risk: all the patients with high risk PE had this presenting symptom (39.4%). Only half of low-risk PE patients complained of dyspnea (27.5%). The incidence of dyspnea was increased with the risk of PE, and this observation was also noticed by Khasin et al. [29].

Right-sided heart failure signs (dilation of jugular veins; cyanosis; tachycardia; hepatomegaly; hepatic jugular reflux; and cardiac edema) were noticed among 40% of the patients. RVD at CTPA (42% of the patients) was concordant with clinical signs of right-sided heart failure (40% of the patients). Wang et al. noticed a heart failure prevalence of 34% among patients with PE [30].

The third part of our patients revealed hemodynamic instability at hospital admission. Other authors reported lower percentages, around 12%, for this condition in PE [31,32]. Our patients had a higher prevalence of hemodynamic instability because ‘’St. Spiridon” Emergency Hospital admits patients living in the second biggest city of our country, Iasi, and in several counties, nearby Iasi.

RVD was a major parameter in PE risk stratification; intermediate- and high-risk PE had this condition. RVD was established by the following CTPA parameters: RV/LV; PAT/Ao; septal deviation; and contrast reflux in the inferior cava vein. The prevalence of RVD among our patients was 42%, comparative with the values around 30% revealed by other authors [33,34].

Comparing our CTPA protocol with those in the literature, we discovered several differences:

- A cranio-caudal thoracic scan was performed in our radiology clinic (depicting better the contrast in the inferior segmental pulmonary arteries) compared with the caudo-cranial scan preferred by Nguyen et al. [35]. This cranio-caudal scan in our protocol prevented the respiratory artifacts in the lower lobes and avoided the artifact from the high-intensity contrast in the superior vena cava.
- Our protocol includes a venous phase. This has the following advantages: seeing the pulmonary arteries twice (in both arterial and venous phases) and resolving some opacification problems given by common physics artifacts and patient characteristics (body habitus, motion artifacts, and cardiac output). The disavantage of venous phase from our protocol is higher radiation exposure for the patient.

We noticed a very strong statistical correlation (Rho values) between PAOI and D-dimer. The entire sample revealed this correlation and also the four groups with different etiologies: DVT, AF, COVID-19, and COPD. Only for cancer patients, the relationship had no statistical significance, and this observation has the following explanation: elevated D-dimer values are usually noticed in cancer patients, without PE association. Elevated D-dimer values above 1128 µg/L were noticed in massive PE. Lower D-dimer values, below 1099 µg/L, were registered in minor PE. These results suggested that D-dimer could be used as a valuable prognostic marker in PE, both in DVT-associated PE and in isolated PE, as their values were correlated with PE severity. This observation is consolidated by Rho values (0.8 ≤ Rho ≤ 1.00) for the correlation between D-dimer and PAOI. PAOI expressed the PE extent and the PAOI–D-dimer relationship had very strong statistical significance, so D-dimer values could suggest the PE outcome. A positive correlation between PAOI and D-dimer values was also noticed by other authors [36].

We investigated the association between PAOI and c-TnT values. Myocardial injury biomarkers (c-TnT) had elevated values correlated with PE severity and extent (PAOI). Rho values for the relationship PAOI–cTnT in the entire sample and in four groups of PE (DVT, AF, COPD, and COVID-19) suggested very high statistical significance (0.8 ≤ Rho ≤ 1.00). These observations proved that c-TnT could be a valuable marker for PE prognosis, as well as D-dimer. Cil et al. noticed that PAOI was higher in the high troponin I group than in normal troponin I group [37]. In our research, only in cancer patients, the association between PAOI and c-TnT had no statistical significance. This observation can be explained by the following: c-TnT is elevated in cancers due to the cardiotoxicity of chemotherapy and tumor secretion products [38], independent of PE association.

For risk prediction in PE, PAOI was a powerful and accurate marker in this study. AUC was 0.993 for PAOI in high-risk PE. This result proved the very high accuracy of PAOI as a valuable risk predictor in PE. The cut-off value for PAOI was 32.5%. All the PAOI values above 32.5% suggested massive PE and high risk. Similar cut-off PAOI values (above 40%) were noticed by Inonu et al. [39].

In this study, accurate predictors for 7-day mortality were both biomarkers: D-dimer and c-TnT. Elevated D-dimer and c-TnT values were statistically correlated with 7-day mortality in the entire sample and in AF, COVID-19, and cancer patients. The following is a peculiar aspect of our research: the investigation of 7-day mortality predictors in PE. Other studies evaluated the 30-day, 90-day, or 180-day mortality in PE, and the authors noticed the same positive correlation between biomarkers (D-dimer and c-TnT) and PE mortality [40,41,42]. Karolak et al. noticed that c-TnT had an independent prognostic value in PE, and cardiogenic shock was more prevalent when c-TnT values were elevated [43]. Conversely, low c-TnT values were associated with low risk of mortality in PE, as was remarked by Giannitsis et al. [44].

In our research, PAOI had statistical significance on 7-day mortality only for COVID-19 and cancer patients. Zorlu noticed that PAOI > 46.2% was an independent predictor for 30-day mortality in PE [45].

This study has the following limitations: surgical patients postoperatively were not included; natriuretic peptides were not measured; and the comparison between RVD on CTPA and RVD on echocardiography was not discussed.

PE is still noticed in surgical patients, even if, postoperatively, anticoagulation is established by guidelines. This situation is ussually observed among orthopedic patients. LMWH or DOAC administration is recommended for 30–45 days after orthopedic surgery. Some of these patients have interrupted anticoagulation and they are complicated with PE, so they can be investigated by researchers as a specific category.

Natriuretic peptides are valuable biomarkers for heart failure. Their determination in PE consolidates heart failure diagnosis. High levels of natriuretic peptides in PE are correlated with imagistic findings of heart failure: low ejection fraction of RV and high RV volumes [46].

RVD is diagnosed by echocardiography and/or CTPA in PE. Septal deviation, RV/LV ratio, and inferior cava vein reflux are echocardiographic, along with CTPA findings. Combination of these parameters increases the sensitivity and specificity [47]. In our study, we preferred only CTPA findings for RVD diagnosis in order to diminish the evaluation time for PE stratification.

Future directions will include also echocardiographic findings for RVD assesement and the comparison between their accuracy and CTPA findings accuracy. Echocardiographic and CTPA findings will be correlated with natriuretic peptide values. A special aspect will be PE after surgery, with the evaluation of peculiarities in different fields: gynecology, orthopedy, and abdominal surgery.

## 5. Conclusions

PE risk and 7-day mortality assesment was the major objective of this study. Imaging, represented by PAOI, was important for risk stratification. A cut-off PAOI value above 32.5% defined massive PE. PAOI was an accurate predictor for high-risk PE. Biomarkers, D-dimer and c-TnT, were accurate predictors for 7-day mortality in the entire sample and in specific PE etiologies: AF, COVID-19, and cancer. PAOI had statistical significance in 7-day mortality only in COVID-19 and in cancer patients. We noticed a very strong correlation between PE extent (PAOI) and biomarkers (D-dimer and c-TnT) for the entire sample and for specific etiologies: AF, DVT, COPD, and COVID-19. Laboratory biomarkers were important tools for 7-day mortality assesment, while PAOI was a valuable predictor for PE risk assesment.

## Figures and Tables

**Table 1:** Characteristics of the sample.

Characteristics	n	%
Gender		
Male	49	45.0
Female	60	55.0
Age (mean ± SD)	66.79 ± 14.017	
Obesity	64	58.7
Dyspnoea	73	66.9
Heart failure clinical signs	44	40.3
Hemodynamic instability	38	34.9
DVT	35	32.1
AF	32	29.4
COVID-19	28	25.7
Cancer	14	12.8
COPD	7	6.4

n = number; SD = standard deviation.

**Table 2:** The correlation between PAOI and D-dimer.

		m ± SD	95% CI	Median	Q1 ÷ Q3	*p*-Value ^†^	Rho ^‡^
Entire sample (n = 109)		D-dimer					
PAOI	<32.5%	835.55 ± 244.533	778.49 ÷ 892.60	798.00	659.50 ÷ 942.00	<0.001 **	0.855 **
	≥32.5%	1376.25 ± 181.981	1314.68 ÷ 1437.82	1387.00	1216.50 ÷ 1527.00		
DVT (n = 35)		D-dimer					
PAOI	<32.5%	782.36 ± 211.364	688.65 ÷ 876.08	794.00	595.75 ÷ 884.25	<0.001 **	0.908 **
	≥32.5%	1327.54 ± 215.926	1197.06 ÷ 1458.02	1257.00	1128.00 ÷ 1525.00		
AF (n = 32)		D-dimer					
PAOI	<32.5%	761.09 ± 223.029	662.21 ÷ 859.98	711.00	581.50 ÷ 906.00	<0.001 **	0.942 **
	≥32.5%	1349.10 ± 165.779	1230.51 ÷1467.69	1372.00	1184.00 ÷ 1456.75		
COPD (n = 7)		D-dimer					
PAOI	<32.5%	788.80 ± 116.395	644.28 ÷ 933.32	823.00	684.00 ÷ 876.50	0.095	0.982 **
	≥32.5%	1313.50 ± 9.192	1230.91 ÷1396.09	1313.50	1307.00 ÷ 1320.00		
COVID-19 (n = 28)		D-dimer					
PAOI	<32.5%	953.24 ± 251.433	823.96 ÷ 1082.51	918.00	728.50 ÷ 1099.50	<0.001 **	0.913 **
	≥32.5%	1472.82 ± 143.500	1376.41 ÷ 1569.22	1493.00	1356.00 ÷ 1552.00		
Cancer (n = 14)		D-dimer					
PAOI	<32.5%	1000.50 ± 286.747	818.31 ÷ 1182.69	923.00	778.75 ÷ 1398.00	0.352	0.091
	≥32.5%	1364.00 ± 48.083	931.99 ÷ 1796.01	1364.00	1330.00 ÷ 1322.00		

^†^ Mann–Whitney test; *p* < 0.01 ** high statistical significance; ^‡^ Spearman’s correlation coefficient; 0.8 ≤ Rho ≤ 1.00 very strong statistical correlation; Q_1_ minimum value; Q_3_ maximum value.

**Table 3:** The correlation between PAOI and c-TnT.

		c-TnT				Total		*p*-Value ^†^	Rho ^‡^
		Normal		Increased					
		n	%	n	%	N	%		
Entire sample (n = 109)									
PAOI	<32.5%	QA	100.0%	19	34.5%	73	67.0%	<0.001 **	0.815 **
	≥35%	-	-	36	65.5%	36	33.0%		
Total		54	100.0%	55	100.0%	109	100.0%		
DVT (n = 35)									
PAOI	<32.5%	19	100.0%	3	18.8%	22	62.9%	<0.001 **	0.882 **
	≥32.5%	-	-	13	81.3%	13	37.1%		
Total		19	100.0%	16	100.0%	35	100.0%		
AF (n = 32)									
PAOI	<32.5%	17	100.0%	5	33.3%	22	68.8%	<0.001 **	0.937 **
	≥32.5%	-	-	10	66.7%	10	31.3%		
Total		17	100.0%	15	100.0%	32	100.0%		
COPD (n = 7)									
PAOI	<32.5%	-	-	5	71.4%	5	71.4%	-	0.982 **
	≥32.5%	-	-	2	28.6%	2	28.6%		
Total		-	-	7	100.0%	7	100.0%		
COVID-19 (n = 28)									
PAOI	<32.5%	13	100.0%	4	26.7%	17	60.7%	<0.001 **	0.828 **
	≥32.5%	-	-	11	73.3%	11	39.3%		
Total		13	100.0%	15	100.0%	28	100.0%		
Cancer (n = 14)									
PAOI	<32.5%	9	100.0%	3	60.0%	12	85.7%	0.110	0.118
	≥32.5%	-	-	2	40.0%	2	14.3%		
Total		9	100.0%	5	100.0%	14	100.0%		

^†^ Pearson chi-squared test; *p* < 0.01 ** high statistical significance; ^‡^ Spearman’s correlation coefficient; 0.8 ≤ Rho ≤ 1.00 very strong statistical correlation.

**Table 4:** PE risk assessment for the entire sample and for different etiologies.

PE Etiology	Low Risk (n, %)	Intermediate Risk (n, %)	High Risk (n, %)
Entire sample	69 (63.3)	4 (3.66)	36 (33)
DVT	22 (20.1)	-	13 (11.9)
AF	22 (20.8)	-	10 (9.17)
COPD	1 (0.91)	4 (3.66)	2 (1.82)
COVID-19	17 (15.5)	-	11 (10)
Cancer	12 (11)		2 (1.83)

**Table 5:** AUC and PAOI cut-off value in high-risk PE prediction.

Area Under Curve	*p*-Value	95% CI				
		Lower Bound	Upper Bound	Sensitivity	Specificity	PAOI Cut-Off Value
0.993	0.000 **	0.983	1.000	100.0%	97.1%	32.5%

*p* < 0.01 ** high statistical significance.

**Table 6:** Biomarkers, PAOI, and 7-day mortality in the entire sample with PE.

Entire Sample (n = 109)	7-Day Mortality		*p*-Value ^†^
	Yes (n = 9)	No (n = 100)	
D-dimer (m ± SD)	1502.78 ± 102.303	970.15 ± 319.202	<0.001 **
cTnT (m ± SD)	134.78 ± 2.279	44.86 ± 46.601	<0.001 **
PAOI (m ± SD)	31.94 ± 27.607	27.06 ± 20.664	0.741

^†^ Pearson chi-squared test; *p* < 0.01 ** high statistical significance; m ± SD = mean ± standard deviation; n, number.

**Table 7:** Biomarkers, PAOI, and 7-day mortality in AF patients with PE.

AF (n = 32)	7-Day Mortality		*p*-Value ^†^
	Yes (n = 2)	No (n = 30)	
D-dimer (m ± SD)	1436.00 ± 8.485	912.10 ± 329.800	0.036 *
cTnT (m ± SD)	133.50 ± 2.121	39.97 ± 46.714	0.004 **
PAOI (m ± SD)	33.75 ± 22.981	26.17 ± 22.008	0.532

^†^ Pearson chi-squared test; *p* < 0.05 * statistical significance; *p* < 0.01 ** high statistical significance; m ± SD = mean ± standard deviation; n, number.

**Table 8:** Biomarkers, PAOI, and 7-day mortality in COVID-19 patients with PE.

COVID-19 (n = 28)	7-Day Mortality		*p*-Value ^†^
	Yes (n = 5)	No (n = 23)	
D-dimer (m ± SD)	1546.00 ± 123.968	1072.87 ± 304.477	0.003 **
cTnT (m ± SD)	135.60 ± 1.517	49.04 ± 50.963	0.002 **
PAOI (m ± SD)	49.50 ± 25.274	26.20 ± 18.963	0.045 *

^†^ Pearson chi-squared test; *p* < 0.05 * statistical significance; *p* < 0.01 ** high statistical significance; m ± SD = mean ± standard deviation; n, number.

**Table 9:** Biomarkers, PAOI, and 7-day mortality in cancer patients with PE.

Cancer (n = 14)	7-Day Mortality		*p*-Value ^†^
	Yes (n = 3)	No (n = 11)	
D-dimer (m ± SD)	1451.00 ± 24.269	943.73 ± 229.250	0.005 *
cTnT (m ± SD)	134.33 ± 3.215	26.36 ± 37.294	0.005 **
PAOI (m ± SD)	7.50	22.95 ± 14.655	0.038 *

^†^ Pearson chi-squared test; *p* < 0.05 * statistical significance; *p* < 0.01 ** high statistical significance; m ± SD = mean ± standard deviation; n, number.

## Data Availability

The original contributions presented in this study are included in the article; further inquiries can be directed to the corresponding authors.
